# The making of a controversy: The Hansen-Neisser debate revisited

**DOI:** 10.1017/mdh.2026.10063

**Published:** 2026-07

**Authors:** Meinrad Pohl

**Affiliations:** Department of Pedagogy, Religion and Social Studies, Western Norway University of Applied Sciences, Bergen, Norway

**Keywords:** *Mycobacterium leprae*, Discovery, Controversy, Bacteria, Leprosy, Source criticism

## Abstract

Literature about the discovery of the leprosy bacterium *Mycobacterium leprae* usually mentions the Hansen-Neisser controversy. It is an established narrative that Neisser tried to present himself as the discoverer of the leprosy bacterium. However, Neisser’s first publication on *Mycobacterium leprae* refers explicitly to Hansen’s original discovery. How did the notion of Neisser trying to claim Hansen’s discovery emerge? A thorough source-critical examination of Hansen’s and Neisser’s scientific and popular publications between 1874 and 1897 as well as of the literature dealing with the so-called controversy brought interesting new results. The notion that Neisser tried to steal Hansen’s discovery is based on a misreading of Neisser’s publications by Hansen’s biographer. But Neisser tried indeed to bypass Hansen in trying to prove that *Mycobacterium leprae* was the contagion causing leprosy. The competition between the two scientists resulted in a personal enmity. This study shows how the history of modern science is prone to myths and underlines the importance of source criticism when dealing with established narratives.

## Introduction

The historical account of the discovery of the leprosy bacterium reads like a classical narrative based on a clear dichotomy of hero and villain. The hero is Gerhard Henrik Armauer Hansen. On the evening of 28 February 1873, he discovered the almost invisible leprosy bacteria after long, strenuous evenings on the microscope, writing down his results and publishing them in modest words. He had advanced in his noble cause to prove that leprosy was contagious and had discovered the probable contagion. The villain is Albert Neisser. He used samples he received from Hansen, stained the bacteria, made them visible to a larger audience, published the results behind Hansen’s back, and claimed to be the discoverer. Seeing his status as discoverer threatened, Hansen deviated from the right path, tried to push things, and experimented on a patient in order to prove contagiousness. The experiment failed, and Hansen was sued and punished for his misconduct. At the same time, a conflict between Hansen and Neisser evolved. This conflict between the two scientists was labelled a struggle for fame and was called the ‘Hansen-Neisser controversy’. But in the end, justice prevailed, and the controversy was put to an end at the first international leprosy congress in Berlin 1897. Hansen was hailed as the discoverer of the leprosy bacterium and thus granted access to the Olympus of sciences.

### A true story?

This slightly exaggerated paragraph reflects a widely accepted narrative about the discovery of *Mycobacterium leprae*, as it can be read in many places.^1^ It has a somewhat hagiographic quality, exhibiting the central elements of a classical heroic tale. The noble hero embarks on a quest and is contested by the villain. In a moment of impatience and desperation the hero deviates from the righteous path, meets his nemesis, is put to justice, and thus back onto the righteous path. Ultimately, his patience and justice prevail, and the hero triumphs. This established narrative has been reiterated so frequently that it has assumed the status of a truth of its own, a reified narrative. But is it true?

Working on an exhibition to commemorate the 150-year anniversary of Hansen’s discovery at the City Museum in Bergen, the curatorial team agreed on presenting this historical account. The section addressing the Hansen-Neisser controversy was set to conclude with the quote from Neisser’s presentation where he claimed the discovery, the actual *corpus delicti.* Then this section could be completed, and the texts sent to print. But no *corpus delicti* could be found, neither in Neisser’s original presentation from 1879 nor in his subsequent publications. Turning from the primary sources to the literature, it became apparent that Hansen attributed minimal significance to this incident in his memoirs. He emphasised his relaxed attitude towards scientific honours and public recognition but still accused Neisser of attempting to bypass him.^2^ While Hansen’s statement remains ambiguous, the biographies explicitly accuse Neisser of seeking credit for Hansen’s discovery and mention a ‘Hansen-Neisser controversy’.^3^

This raises important questions: How can we account for the disparity between the primary sources and the biographies? From where did this narrative emerge and how did it acquire factual status? Having examined the relevant literature, alongside the publications of Hansen and Neisser it became evident that while there indeed was a conflict between the two scientists, it did not centre on the actual discovery of the bacterium. The objective of this article is to examine this so-called controversy and the narrative that has surrounded it. In doing so, this article seeks to challenge older hagiographic historiography by placing science and scientist within the historical framework as described by Shapin.^4^ It is a contribution to the field of scientific controversies as examined by Engelhardt and Caplan, adding to it as a case study of a misconceived controversy.^5^

The discovery of *Mycobacterium leprae* is central to both the history of leprosy and the broader history of medicine. In the latter half of the nineteenth century, leprosy was among the diseases researched to establish their bacterial origin, a concept that had not yet been proven. The discovery of *Mycobacterium leprae* altered the understanding of leprosy, framing it as a contagious disease rather than a hereditary one, although definitive proof of its contagiousness could not be established in the nineteenth century. Understanding the nature of the disease was essential in addressing leprosy as a public health issue and developing a cure. The narrative of the Hansen-Neisser controversy plays an integral role in the history of the discovery of *Mycobacterium leprae.* Besides setting the record straight, unveiling it as a myth is both a caveat and a reminder of the truism to always consult the primary sources in historical research.

Prior to presenting the problems and methodologies of this investigation, it is essential to provide background context and elucidate how the narrative about the controversy has evolved. Following this, the methods employed will be described, and the narrative on the controversy as it has emerged in literature will be critically assessed against the primary sources.

### The making of a controversy

In his memoirs, Hansen offers a brief account of the encounter with Neisser. Hansen writes that he failed in staining the bacteria using Weigert’s and Koch’s methods. When Neisser visited him, Hansen hoped that a physician from the environment of Weigert and Koch could help him stain the bacteria. However, Hansen was disappointed to find that Neisser could not help him, according to Hansen, because he was no more able than Hansen himself. He provided Neisser with samples, who succeeded in staining them when back in Breslau. Nevertheless, Hansen asserts that he also managed to stain the bacteria in the meantime, aided by advice from Koch in a letter. Hansen writes that he felt no urge to publish his result, contrary to Neisser. He acknowledges Neisser’s decency to mention what he had demonstrated to Neisser and continues: ‘I took this attempt to bypass me very calmly’. Characterising himself as someone who ‘was never suffering from any real vanity or personal ambition’, Hansen attributed all anger regarding Neisser’s behaviour to his superior, Danielssen, who accused Neisser of attempting to steal Hansen’s discovery. But Hansen also admits that he found it worthwhile to publish his results also in a German journal.^6^

In 1917, the German physician Czaplewski published a rather polemic article on the occasion of Neisser’s death, attributing the honour for the discovery solely to Neisser, downplaying the importance of Hansen’s discovery. Czaplewski sees first Neisser’s staining and demonstration of the bacteria as the proper proof and discovery of the leprosy bacterium, arguing that Hansen first published his results after Neisser, thus ignoring Hansen’s original publication from 1874.^7^

In 1955 the American physicians and specialists in leprosy, Fite and Wade, published an article on the controversy and examined Neisser’s role. They explicitly state that Hansen made the original discovery and highlight that the actual question is more about how Neisser had contributed in establishing the bacterium as the aetiological agent.^8^ They criticise Neisser’s 1879 publication for discrediting Hansen and spending much effort to assert the importance of his own work instead of giving Hansen any credit, and attempting to erase Hansen from the picture even before he came in.^9^ They write that Neisser bore resentment because he felt he had not received due recognition. They also point out a personal enmity, writing ‘The matter engendered some heat between the two men, but only between them’.^10^ They argue that Neisser’s demonstrations were the first satisfactory evidence of the connection between the bacteria and the disease, but also that satisfactory techniques were not available at the time of Hansen’s discovery. Furthermore, they dismissed the importance of Hansen’s 1880 article, attributing this to his inactivity between 1874 and 1879. They conclude that the difference in Hansen’s and Neisser’s approach was that Hansen was interested in leprosy in general, the aetiology just being one aspect, while Neisser was interested in micro-organisms causing disease, with leprosy being just one case.^11^ They do not really shed light on the controversy as their analysis shifts the focus from the original discovery to proof of the bacterium as the causative agent. But they fail to reach a decisive conclusion on this topic.

In 1963, Vogelsang expanded on Fite and Wade’s article detailing Hansen’s significant scientific contributions leading to the discovery.^12^ With reference to Hansen’s 1874 article, he argues that although Hansen was convinced that the bacteria were the causative agent, he recognised that he lacked evidence to prove it. Vogelsang confirms Fite and Wade’s statement that Neisser’s 1881 publication was ‘an outstanding definitive description of the relation of the bacilli to the lesions and of their etiologic import’.^13^ He also emphasises Hansen’s busy career as a practising physician and government official, refuting claims of inactivity after his 1873 discovery.

In his Hansen biography, published in Norwegian in 1968 and translated into English for the *International Journal of Leprosy* in 1978, Vogelsang devotes a chapter to the so-called Hansen-Neisser controversy, mainly based on Fite and Wade’s article. He writes expressively about ‘the controversy’ and reproduces Hansen’s account about the events. Building on Fite and Wade, Vogelsang expresses that the credit for the original discovery without any doubt belonged to Hansen.^14^ Yet, Vogelsang’s reproduction of Neisser’s 1881 article implies that Neisser indeed claimed the fame for the discovery.^15^ He even claims that some German scientists tried to call the leprosy bacterium *Neisser’s bacillus*, although he provides no references to substantiate this assertion. He also states quite clearly that, ‘Already at the First International Scientific Leprosy Conference in Berlin in 1897 he [i.e. Hansen] was, without opposition from anyone declared the discoverer of the leprosy bacillus which was regarded as the etiologic agent of leprosy’, and that the Hansen-Neisser controversy had already ‘sunk deep into oblivion’.^16^

In the Hansen biography by Patrix, Hansen’s granddaughter, the incident with Neisser is only mentioned in the epilogue written by Irgens. Also, Irgens writes about a controversy over the discovery of the leprosy bacterium, stating that Neisser claimed to be the discoverer of the leprosy bacterium in his 1879 article and characterising Neisser’s publications as the ‘theft of his outstanding scientific achievement’.^17^ Irgens, a student of Vogelsang, claimed this first on a conference in 1970, relying on Vogelsang’s Hansen biography and his translation of Neisser’s 1881 article, subsequently repeating this assertion in various publications.^18^

All the aforementioned publications were written by physicians, and the topic was only recently covered by historians. In his master’s and PhD theses, which both refer to the controversy albeit not as the central focus, Vollset more or less reproduces Vogelsang’s position about the Hansen-Neisser controversy and the claim that it was decided in Hansen’s favour on the first leprosy congress in Berlin in 1897.^19^ He hints at discrepancies in the literature regarding the intensity of the conflict, particularly concerning Neisser’s claim for priority. But he only refers to the above-mentioned literature^20^ and does not delve into greater detail.^21^

In his PhD thesis Bechler approaches the controversy with a different perspective on the term ‘discovery’. He does not define ‘discovery’ as the first observation and description of the bacterium, but as the successful establishment of the bacterium as aetiological agent. Hence in this case this addresses the later competition between Hansen and Neisser to do so. Bechler is therefore more interested in this process.^22^ Bechler suggests that Hansen willingly provided Neisser with samples to present himself as generous, to test Neisser’s capabilities, and to expose him as unable should he fail.^23^ Bechler agrees with Fite and Wade regarding Neisser’s publication as the first academically acceptable description of the possible aetiological agent. He also claims that Neisser succeeded before Hansen to test the preparates for contagiousness.^24^ Bechler also investigates the role of Robert Koch, the role of Rudolf Virchow, the editor of the influential journal *Archiv für pathologische Anatomie und Physiologie und für klinische Medicin* [Archive for Pathological Anatomy and Physiology and Clinical Medicine], later renamed *Virchows Archiv*, and the role of Hansen’s and Neisser’s publications.

The established narrative describes Hansen as the original discoverer of the leprosy bacterium and also seems to align with Hansen’s memoirs that state that Neisser attempted to bypass him. However, both the memoirs and the later narrative are unclear about what this bypassing entailed. Did ‘bypass’ mean that Neisser tried to present himself as the original discoverer of *Mycobacterium leprae* as Vogelsang and Irgens put it – bypassing in terms of discovery? Or did it refer to Neisser’s efforts to establish the bacterium as the actual contagion like Bechler suggests – bypassing in terms of proof? The end of the narrative becomes clear again when asserting that the Berlin conference of 1897 resolved the debate in favour of Hansen. Rarely explicit but often between the lines, also a personal enmity between the two researchers becomes increasingly evident.

### Problem and methodological approach

In this article, I will explore three aspects of the conflict-laden relationship between Hansen and Neisser. The first two aspects are commonly labelled the Hansen-Neisser controversy. The first aspect is the actual controversy about who had discovered the leprosy bacterium, as claimed by Vogelsang. The second, as argued by Bechler, is that it actually had been a rivalry to be the first to prove that leprosy was contagious. The third aspect is the personal enmity between Hansen and Neisser at which Fite and Wade hinted.

To examine these three aspects, I will analyse a corpus of Hansen’s and Neisser’s publications on *Mycobacterium leprae* from 1874 to 1897. The corpus begins with Hansen’s original publication from 1874, marking the first major occurrence of the bacterium in a publication, and concludes with the conference proceedings of the first international leprosy congress held in Berlin 1897, which is ascribed credit as the forum resolving the controversy.

#### Controversy – a fight for priority?

To address the first aspect regarding the controversy – referred to as ‘the bypassing in terms of discovery’ – I will evaluate the narratives presented in the biographies against the aforementioned corpus. I will scrutinise the articles to determine who claimed the discovery of *Mycobacterium leprae.* In this context ‘discovery’ refers to the first observation of the bacteria under a microscope and the subsequent description in a scientific publication. The approach will follow classical source criticism.

#### Rivalry – the competition for proof

To explore the second aspect, the rivalry to prove the bacterium’s contagiousness – referred to as ‘the bypassing in terms of proof’ – I will analyse the same publications in the light of the so-called Koch postulates. Since time is crucial for this kind of competition, I will assess the articles according to their plausibility concerning the chronology of events. Although the postulates were not formulated in their final form at the time of the discovery, their claims were known and referred to, making them a suitable methodological framework. I will examine whether Hansen and Neisser succeeded in

- proving the existence of *Mycobacterium leprae* in skin nodules and nerves in both forms of leprosy, macular and anaesthetic
- growing *Mycobacterium leprae* in pure laboratory culture
- transferring the disease to a new host, animal, or human.

While the last two postulates are relatively straightforward, the first postulate contains several criteria. For this article, I will investigate whether the first postulate was fulfilled in a necessary and sufficient manner. Here, ‘necessary proof’ means that all the observed leprosy bacteria derive from cases of leprosy and their products and are hence connected with the disease and not secondary epiphenomena. ‘Necessary and sufficient proof’ means that the bacteria had also been proven in both forms of leprosy – macular and anaesthetic.

To accomplish this, it will be necessary to incorporate some modern knowledge about the aetiology of leprosy. We now know that *Mycobacterium leprae* does not produce spores. A successful cell culture was first achieved in 2018.^25^ In addition to humans known hosts include certain species of armadillos, as documented in wildlife studies and experimental inoculation,^26^ and some primates, first identified as hosts in the latter half of the twentieth century.^27^ More recent wildlife studies have reported *Mycobacterium leprae* infections in red squirrels, as well as isolated cases in lowland tapirs and margays.^28^ Experimentally, leprosy has been grown in the footpads of mice and, more recently, in zebrafish.^29^ Additionally, *Mycobacterium leprae* has been shown to survive in amoeba, kissing bugs, and ticks.^30^

#### Enmity – the clash of egos

Thirdly, I will investigate whether the articles of the corpus provide some insight into the personal enmity between Hansen and Neisser. The approach will again follow classical source criticism.

## The controversy – found in translation?

After examining Neisser’s publications, it is safe to conclude that Neisser had made no attempt to claim Hansen’s discovery for himself. In his original presentation from 1879, and his subsequent publications from 1881 and 1883, Neisser explicitly refers to Hansen and his discovery, possibly to ensure he was not seen as claiming the original discovery. However, as Neisser states himself in 1881, he claims to have proven that the bacteria were the contagion.^31^

As previously mentioned, Hansen devoted less than two pages of his memoirs to the incident with Neisser. He wrote that he took Neisser’s attempt to bypass him very calmly, but that Danielssen was outraged and accused Neisser of trying to steal Hansen’s discovery.^32^ The meaning of ‘bypass’ remains ambiguous.

The matter was brought up again by Czaplewski in 1917, but his article gained limited attention, until Fite and Wade revisited it to discuss the role Neisser had in establishing the leprosy bacterium as the aetiological agent.^33^ Although Fite and Wade claim in their headline to deal with the so-called Hansen-Neisser controversy and call the controversy most unfortunate, they conclude with ‘The vast bulk of the literature of leprosy is devoid of suggestion of controversy in this matter’.^34^ They state clearly that the credit for the original discovery goes to Hansen and do not even address whether Neisser tried to claim the discovery. While Fite and Wade give Neisser a certain role in establishing the leprosy bacterium as the aetiological agent, Vogelsang emphasises Hansen’s role.^35^

The publications of Fite and Wade and Vogelsang contribute more to the notion of a controversy than they resolve the issue, and so does Vogelsang’s Hansen biography. As a biographer Vogelsang sides with Hansen, mentioning a controversy in the years 1879–1881 and claiming that some German scientists attempted to name the bacterium after Neisser, without giving any references to support this assertion. Furthermore, he works with extensive quotations, including Neisser’s 1881 article which he reproduces with an incorrect translation. Neisser’s original words are:

Ich vindicire mir dagegen das Verdienst, diesen bis dahin (…) von Niemand beachteten Mikroorganismen ihre Stelle unter den pathogenen Pilzen gesichert zu haben,…^36^

A correct translation would be ‘I vindicate [which means here: claim, defend for myself] the merit to have secured a place among the pathogenic fungi for these micro-organisms that had not been recognised by anybody’ [which means: neglected by all].

For his Hansen biography published in Norwegian in 1968, Vogelsang translates Neisser’s statement with the words:

Jeg ønsker å ha æren for å ha gitt disse mikroorganismer, som ingen før den tid hadde sett, en plass blant de sykdomsfremkallende fungi,…^37^

For the English version of the biography published in 1978, he translates his own Norwegian translation into English, which reads:

I wish to have the honor of having given this microorganism, which no one before that time had seen, a place among fungi calling forth disease…^38^

Vogelsang simply alters Neisser’s statement that the micro-organisms discovered by Hansen were widely neglected into a claim that nobody had observed them before! The wording of Vogelsang’s translation of Neisser’s article might very well have fostered the impression that Neisser indeed attempted to claim the credit for the original discovery. He aligns with Hansen’s description of Danielssen’s reaction.

Based on Vogelsang’s incorrect translation, his student Irgens states in 1970 explicitly that Neisser wrote:

… å få æren for å ha gitt disse mikroorganismer som ingen før hadde sett, en plass blant de sykdomsfremkallende fungi.^39^

This translates to English as: ‘To get the honour to have given these micro-organisms that nobody had seen before a place among the pathogen fungi’. Irgens even shortens the quote in a manner that makes the suggestion of Neisser’s claim of being the original discoverer even more clear. Over more than 50 years, Irgens repeated in various publications that Neisser had claimed the fame for the discovery, sometimes referring to Vogelsang, sometimes referring to Neisser’s original article.^40^ Language barriers appear to significantly influence this discourse, as none of the mentioned scholars seemed to possess sufficient proficiency in Norwegian, German, and English to be able to accurately engage with the original sources or to provide correct interpretations and translations of them. This confusion has led to an academic rumour that acquired factual status. Another albeit less severe example for incorrect translations, or rather a misreading, occurs when Fite and Wade quote Hansen’s memoirs suggesting that he wrote his 1880 publication in *Virchows Archiv* upon invitation.^41^ This phrasing is not found in Hansen’s memoirs or articles; he merely stated that he deemed it worthwhile to publish his results in a German periodical.

Vogelsang claimed that the international conference on leprosy in Berlin 1897 had resolved the debate over who discovered the leprosy bacterium, yet the question of discovery was not a topic of discussion. While there was no formal resolution awarding the discovery of the leprosy bacterium to Hansen, the proceedings of this conference leave no doubt that Hansen was accepted as the discoverer. Summarizing the results of the congress, Bergmann, translated by Abraham, stated introductorily:

As might be expected, a considerable portion of the discussion has related to the Bacillus Leprae, which the Conference accepts as the Virus of Leprosy, and which for upwards of 25 years has been known to the scientific world through the important discovery of Hansen and the able investigations of Neisser.

The remainder of this summary focusses on the still unknown lifecycle of the bacterium, the high probability of its contagiousness, and the practical implications to conclude with the recommendation of the isolation of the infected.^42^

However, most contributors who were touching on the discovery at all attributed it to Hansen. The contributions on French, in particular, referred to the bacterium as *‘bacille de Hansen’*, as did some sporadic contributions in English, Spanish, and German. Occasionally, the bacterium was also referred to as ‘*Hansen-Neisser-bacterium’*, also mostly in contributions in French language. Even Neisser himself acknowledged in Berlin, as in previous publications, that Hansen ‘had described the rod-shaped micro-organisms which he could assume were bacteria’. Yet, he still stressed the importance of his own work, when stating that since his publication in 1879 there was no case of leprosy where the bacterium had not been recorded.^43^ His insistence on the importance of his own work appears almost desperate. When talking about the discovery at the conference, Virchow, the great authority in pathology of his time, did not mention Neisser at all.^44^ Ultimately, the Berlin conference did not settle the question about who discovered the leprosy bacterium; that issue had already been settled. Hansen’s appointment as vice president of the Berlin conference further underscores his authority by the time of the opening of the conference. Also, the assertion that Neisser was acknowledged at the leprosy conference in Berlin in 1897 as the one who had confirmed Hansen’s discovery is unfounded, as no such statement appears in the conference proceedings.

After examining the sources, it is evident that Neisser never claimed the discovery of *Mycobacterium leprae.* Consequently, the Berlin conference did not play a decisive role in resolving the controversy either, contrary to claims found in the literature. The general perception of a controversy about the credit for the original discovery of the leprosy bacterium seems to originate from an incorrect translation of Neisser’s 1881 article, first published by Vogelsang, repeated on several occasions by Irgens, and reiterated in several publications by him and others, and finally acquired factual status.

Nevertheless, Hansen’s 1880 publication and the subsequent publications of both Hansen and Neisser on the topic are most interesting, as they indicate that the conflict between them was not about the first observation of the bacterium but rather about the competition to provide proof that the bacterium is the contagion and causative agent of leprosy. This topic will be explored in depth in the next section, titled ‘Rivalry’. Furthermore, this competition seems to mark the beginning of a personal enmity between Hansen and Neisser, which will be addressed in the section titled ‘Enmity’.

## Rivalry

Even though there was no controversy about who had observed the leprosy bacteria first, there was a competition between Hansen and Neisser to prove that the bacterium was the contagion. This part of the relationship between Hansen and Neisser seems not to have gained much attention in the literature on the topic. However, Bechler has analysed this part in some detail in his PhD thesis. Redefining the term ‘discovery’ as ‘sociologic construction, consisting of scientific and personal animosities’, so he, in fact, labels the competition for finding proof for contagiousness as the process of the actual discovery. I will not follow this definition but try to describe the rivalry between Hansen and Neisser, the competition for proof. Hansen’s scientific goal was to prove that leprosy was contagious and not hereditary. At that time, contagiousness was a hot topic and also Neisser wanted to participate in the field of microbiology, but for him, leprosy was just one of many cases.

### How to prove contagiousness? Bacteria as a cause for disease

To understand the issues of Hansen’s and Neisser’s rivalry, it is necessary to consider the historical context in which this rivalry evolved. Therefore, I will outline how contagiousness of a bacterium was proven and how this technique was developed. In 1873, when searching for the contagion that causes leprosy, Hansen was operating on uncharted terrain. Never before had a bacterium been proven to be the cause of a disease, let alone a chronic disease. Louis Pasteur’s research had suggested a correlation between micro-organisms and disease. Davaine had shown in 1863 that the anthrax bacterium, which was first discovered by Pollender in 1849, always accompanied the symptoms, thus making a correlation plausible.^45^ Then, in 1876 Robert Koch succeeded in proving that the bacteria were the causative agent of the disease.^46^ This was the first case when a bacterium was proven to be the cause of a disease. It was a long way from discovering the existence of a bacterium in cases of disease to actually prove that it caused the disease.

Koch also showed the correlation between bacteria and disease for six different forms of septicaemia in 1878 and for tuberculosis in 1882.^47^ Tuberculosis was thus the first chronic disease for which a bacterial cause could be proven.

The suggestion that bacteria caused disease initially provoked scepticism. To meet this scepticism a method, a set of rules, needed to be developed to prove the correlation. While working on the different bacteria, Robert Koch developed such a set of rules, which later became known as the Koch postulates, Henle-Koch postulates, or Koch-Loeffler postulates. For convenience, they will be referred to as Koch postulates for the remainder of this article.

The wording was established by Loeffler in 1883 and can be paraphrased as follows:

1. The micro-organism must be found in all organisms suffering from the disease.
2. The micro-organisms must be isolated from a diseased organism and grown in pure culture.
3. The micro-organisms, grown in pure culture, must be able to cause the disease.^48^

Even though these rules were first formalised as postulates in 1883/1884, the principles had been known and in use several years before. Henle had previously written in 1840 that it would be necessary to isolate micro-organisms and study their properties to prove their role as contagion.^49^ In 1877, Klebs formulated the three steps: isolate, cultivate, inoculate.^50^ Koch repeated these rules in an article about the aetiology of tuberculosis in 1884.^51^ Hansen was also familiar with these rules and worked by them, as evidenced by his letter of defence from a lawsuit against him in 1880.^52^

### The rivalry – who succeeded?

When Hansen and Neisser worked on leprosy, two forms of leprosy were distinguished: the macular and the anaesthetic form. The distinction was primarily based on whether leprous nodules developed on the skin. If that was the case, it was called the macular form. Apart from the skin, internal organs and the nerves were also affected. The anaesthetic form primarily affected the nerves, rendering the affected body parts insensitive, but left the skin mostly intact apart from a rash that left differently pigmented scars after healing. Hansen stresses the difficulty of proving the bacteria in the nerves in anaesthetic cases. Moreover, samples could only be extracted from deceased patients, and by this time the leprous infection was over.^53^ Even though this classification is now abandoned, we need to examine Hansen’s and Neisser’s attempts to prove the postulates for both forms to determine if they had been proven successfully by the standards of their own time.

Hansen had observed the presence of the discovered micro-organisms in all his examined cases of leprosy. This was the first step in proving the bacterium to be the contagion, which Hansen documented in his publication from 1874. However, Hansen had only examined nodules, which implies that he had only observed bacteria in the macular form. This means that he had proven the first Koch postulate only in a necessary but not a sufficient way. Hansen’s mention of failed animal experiments shows that he tried to fulfil the third Koch postulate, but without success.^54^

Hansen’s problem was the insufficient staining technique he had applied in 1873. It took a trained eye and knowledge of what to look for when the preparation was fresh. In the course of a few days, it darkened, making it impossible to see anything. According to Hansen’s 1880 article, Koch’s publication about septicaemia had inspired him to resume his work on the bacteria again in the summer of 1879. Hansen tried to apply Koch’s staining technique, failed, and turned to Koch for help.^55^ In a letter dated 20 July 1879, Koch gave Hansen some advice for staining and asked for specimens for his own research.^56^ This coincided with Neisser’s visit to Bergen in July/August 1879 during his trip to Norway to study leprosy. Hansen showed Neisser his specimens that did not convince the latter. They apparently also tried to stain some samples together but did not succeed. Hansen supplied Neisser liberally with tissue samples before he left Bergen.

Back in Breslau, Neisser succeeded – apparently to everyone’s surprise including his own – quite rapidly in staining the bacteria. He presented the results to his peers on 3 September 1879 and to a larger audience during a meeting of a local scientific society on 10 October 1879. The presentation was published in a local medical periodical in two tranches on 25 October and 8 November 1879. Neisser sent an offprint to Hansen, which he expected to arrive in Bergen on 24 October 1879.^57^

Thanks to his successful staining of the micro-organisms, Neisser was able to prove that they were bacteria and to demonstrate them also in internal organs of infected persons, thus providing a much more elaborate description than Hansen in his original article. However, Neisser too had only observed the bacteria in the macular form and not in the anaesthetic form. Therefore, he also had fulfilled the first Koch postulate only in a necessary but not sufficient way.

Hansen seems to have been taken by surprise by Neisser’s publication. In the letter from 20 July 1879 Koch had assured Hansen not to publish results before Hansen.^58^ Given that at least some of the samples Hansen gave to Neisser were meant for Koch, Hansen had reason to assume that Koch’s assurance would also apply for Neisser. Even if that was not the case, Hansen had reason to assume that Neisser, being part of Koch’s scientific environment, would follow the same rules of conduct as Koch.

With his 1879 presentation, Neisser clearly took the initiative from Hansen, who accepted the challenge immediately. Hansen seems to have started writing his 1880 article – signed Bergen, October 1879 – right after he received the offprint of Neisser’s presentation. This swift response and the fact that Hansen published this article in four languages (Norwegian, German, English, and French) show that Hansen did not take Neisser’s publication lightly, despite how he presented this later in his memoirs.^59^ In these publications Hansen admits that he wrote them to claim priority, which seems understandable. Hansen’s 1874 publication, written in Norwegian, could reach a Scandinavian readership. His English publication of 1875 was only a summary.^60^ Neisser’s publication could reach an audience also in parts of Europe where German was the predominant scientific language at the time including Scandinavia and Eastern Europe. Neisser responded to Hansen’s claim for priority, stating that he thought he had already secured him this priority in two paragraphs in his short presentation in 1879.^61^ Hansen’s description of his findings – all from macular cases – is more detailed than in his 1874 paper, but still less elaborate than Neisser’s and in relation to the first Koch postulate, he made no further progress.

Neisser’s article possibly also spurred Hansen to conduct an experiment on a patient. On 3 November 1879, Hansen pierced the conjunctiva of the left eye of the patient, Kari Nielsdatter Spidsøen, who was suffering from the anaesthetic form of leprosy, with a cataract needle that had material from a leprous nodule on it. The experiment resulted in a lawsuit against Hansen. In his letter of defence, Hansen wrote that he attempted to transfer the macular form of leprosy to a patient who suffered from the anaesthetic form. He quotes a not-preserved letter from Koch that suggested inoculation experiments on animals and humans – possibly already leprous – to rule out the possibility that the observed bacteria were only secondary epiphenomena.^62^ In the previously mentioned letter from 20 July 1879, Koch advised Hansen to be careful to rule out secondary epiphenomena.^63^ The experiment on Spidsøen can be interpreted as an attempt to fulfil the third Koch-postulate for the macular case, aiming for a sudden victorious conclusion to the competition. However, the experiment failed, Hansen was sued, lost his job at the leprosy hospital, and still had not made any further progress.

In his 1881 article Neisser extends his findings to other organs, and particularly important, the nerves. However, he did not specify whether he had observed bacteria in the nerves in a macular or anaesthetic case, thus not advancing in fulfilling the first Koch postulate. Neisser claims to have cultured the bacteria and to have observed spores.^64^ However, *Mycobacterium leprae* does not produce spores and Neisser had to admit in 1883^65^ and 1897 that he was wrong.^66^ Neisser’s animal inoculating experiments also remained without results, even though he believed he had succeeded in one case.^67^

In an attempt to fulfil the second Koch postulate also Hansen experimented with cultivating the bacteria. In his 1882 publication, he claimed to have observed spores during his attempts to cultivate the bacteria in human blood serum.^68^ Hansen thus confirms Neisser’s findings of spores and claims to have extended Neisser’s results.

In *Ziemsens Handbuch der Hautkrankheiten* [Ziemsens handbook of skin-diseases], published in 1883, and translated into English in 1885, Neisser did not distinguish between the macular and anaesthetic forms as was usual, but tried to classify types of leprosy regarding the affection of the skin (*lepra cutanea*) and nerves (*lepra nervosa*). Was this an attempt to sidestep the necessity of demonstrating the bacteria in the anaesthetic form to fulfil the first postulate in a sufficient way? Following this classification, Neisser’s observation of *Mycobacterium leprae* in the nerves in his 1881 article, which most probably derived from a macular case, would have proven the first Koch postulate for *lepra nervosa*, rendering proof for the anaesthetic form unnecessary. In the German text, Neisser claims to have proven Hansen’s assumption, but does not specify whether this is the assumption of the micro-organism being bacteria or being the actual contagion. The English text simply mentions Hansen’s observations and the ‘proof that was still lacking’. Neither the German nor the English text clarifies what Neisser believes he has proven. By staining, Neisser without doubt proved the rod-shaped bodies to be bacteria, but not Hansen’s assumption that the bacteria were the actual contagion.^69^ To do so, it would have been necessary to fulfil all the Koch postulates.

As far as Hansen and Neisser were concerned, the competition ended in a tie: all experiments with cultivation and inoculation had failed; reports of success must be attributed to wrong observations. Progress in fulfilling the first Koch postulate in a necessary and sufficient manner seems to have stagnated after Neisser’s 1881 article. Both Hansen and Neisser only showed the bacteria’s presence in the macular form, thus could only prove the first Koch postulate in a necessary but not sufficient way. In 1884, Arning was the first to observe the bacteria in the nerves in the anaesthetic form and thus succeeded in proving Koch’s first postulate in a necessary and sufficient way.^70^ This remained the case through 1897 and beyond. As Virchow summarised, the bacterium was unanimously accepted as the contagion causing leprosy, even though the bacteriological proof was lacking.^71^

The role of Robert Koch and the publications of both scientists during the rivalry were covered by later historiography, and their accounts are worth scrutinising. Bechler examined the role of Robert Koch, describing his assistance as invaluable to Neisser. He suggests that Hansen first sought Koch’s advice after Neisser had left Bergen, realising Neisser had gained a head start. Bechler argues that Neisser benefited from being located close to Koch, while Hansen had to wait for a reply.^72^ However, it is unclear why Bechler assumes this timeline, as Hansen sought Koch’s advice much earlier, at the latest during Neisser’s visit to Bergen, imprecisely dated to July/August 1879, or even before.^73^ Thus, Koch supported both researchers, not just Neisser.

During the competition, publications played a crucial role. Bechler describes the publication history as a complex interplay of patriotism and personal animosities. He characterises Neisser’s publication in the *Breslauer Aerztliche Zeitung* [Breslau medical newspaper] as a tactical mistake. Although he lacks sources to support this claim, Bechler speculates that Neisser tried to publish his first article in *Virchows Archiv* but was denied due to Virchow’s personal issues with Koch, to whose environment Neisser belonged. Hansen’s memoirs state that Neisser’s article angered Danielssen, Hansen’s superior. So, Bechler concludes that Danielssen possibly had patriotic reasons to support Hansen – despite their own differences – in publishing in Virchow’s influential journal. Bechler also valuates Virchow’s good relationship with Danielssen over any patriotic motives. He does not address why a good standing with Koch only was detrimental for Neisser but not for Hansen. Bechler overrates the role of nationality and patriotic feelings, attributing the posthumous support of Hansen’s role as discoverer by the physicians, Wade and Feldman, to their supposed Norwegian nationality.^74^ Both were US citizens with no apparent ties to Norway. However, when claiming that the publication in *Virchows Archiv* was crucial for Hansen’s reputation as discoverer of the leprosy bacterium, Bechler has a point. *Virchows Archiv* reached a larger audience than the *Breslauer Aerztliche Zeitung*, even though Neisser’s article was not published in its very first issue, as Bechler erroneously states.

Neisser’s publication in the *Breslauer Aerztliche Zeitung* was not necessarily a mistake, if he wanted to present his results to his peers quickly. The text of his presentation from 10 October was published only fourteen days later, while Hansen’s response took two and a half months to be published in *Virchows Archiv.* Possibly, the matter was not suited to attract much attention either. At that time, there were only a few cases of leprosy in the peripheric Memelland area, and colonies had not yet been acquired. Therefore, leprosy was not a major public health concern in Germany. Neisser delivered his presentation in Breslau, and it was published in two parts in a non-prominent place in a local medical periodical. It seems that Neisser, then a young assistant physician, wanted to convince the environment of Koch and the University of Breslau of his scientific capabilities and thereby build his scientific credibility.

Bechler attests to Neisser a couple of mistakes and supports Fite and Wade’s assertion that he wrote offensively about Hansen and Danielssen and their differing opinions on the nature of leprosy.^75^ Upon examining the sources, I cannot find any support for this claim. Bechler considers Hansen’s behaviour during the publication process as tactically smart, in not showing emotions, excitement, or anger and always presenting himself as indifferent to academic honours. Bechler convincingly unveils this as masquerade and assumes that fame was indeed important to Hansen. He reasonably suggests that Hansen was confident in his role as discoverer and that all articles following his 1874 publications had to confirm this role. Bechler assumes Hansen’s reason in providing Neisser with samples was an opportunity to test Neisser’s abilities and to expose him as unable should he fail.^76^ The extent to which Hansen attempted to do the latter will be discussed in detail in the following section.

## Enmity – Do not disturb my circles!

Hansen’s response, spurred by Neisser’s original publication, seems also to have ignited a personal antagonism between the two scientists. After carefully examining the sources, a picture of Hansen and the enmity between him and Neisser emerges that starkly contrasts with the description by Fite and Wade in 1955. They state that only Neisser exhibited some rancour in his 1881 article while Hansen merely touched upon the issue in his memoirs.^77^ However, Hansen’s publications contain significantly more about the matter. The notion that Hansen reacted calmly to Neisser’s publication could already be dismissed. Furthermore, Hansen’s attacks on Neisser directly contradict both his own assertions and Fite and Wade’s statement that Neisser had to ‘find himself sorely abused by Hansen, who had indeed abused him not at all’.^78^ The findings rather support Bechler’s assumption that Hansen wanted to expose Neisser as unable. This starts with Hansen’s 1880 article, in which he replied to Neisser’s article of 1879. In a footnote, Hansen insinuates that Neisser’s success in staining the tissue samples solely depended on the supervision of Koch. He concludes his article with the remark that he, too, only succeeded in staining the bacteria thanks to Koch’s guidance.^79^ In other words, he implies that both he and Neisser succeeded at approximately the same time and that only thanks to Koch’s support. In a later publication, Hansen reiterates this claim asserting that his accomplishments occurred independently from Neisser.^80^ If one accepts Hansen’s assertions, he succeeded in staining the bacteria in late October 1879, but if this can be considered approximately the same time is subject to interpretation. However, this footnote can hardly be interpreted differently than that Hansen tried to downplay Neisser’s achievement in staining the micro-organisms and thus proving them to be bacteria.

Hansen’s efforts to downplay Neisser’s achievements appear to go even further. What initially seems to be casual remarks in a footnote and a postscript can be interpreted as a deliberate attempt to question Neisser’s scientific abilities and his capability to conduct independent research. This undertone surfaces repeatedly in Hansen’s subsequent publications when referring to Neisser’s work. The pattern and the roles are quite clear. Provoked by Neisser’s attempt to take the initiative from him, Hansen attacks Neisser while sowing doubt regarding his scientific capabilities. He refutes Neisser’s results of animal experimentation stating that common sense could have foretold those outcomes.^81^ He criticises Neisser for failing to clarify whether his bacteria findings from nerves derive from macular or anaesthetic cases, to proceed to devaluate his findings referring to prior studies on nerve affections.^82^

Neisser had to admit his own errors, while Hansen busied himself to point out Neisser’s errors, omitting his own misinterpretation of spores, attributing them solely to Neisser,^83^ and even reproaching him for using colloquial terms.^84^ Neisser consistently responds promptly, always positioning himself as the aggrieved party.

The nature of their personal animosity is most evident in Hansen’s criticism of Neisser’s chapter in Ziemsen’s handbook and Neisser’s subsequent reply. In a journal where Neisser was member of the editorial board, Hansen categorically denounces Neisser’s deviating classification of leprosy forms as wrong and absolutely unqualified for a textbook and accuses Neisser of lacking knowledge from his own observation of the anaesthetic form of leprosy, as well as familiarity with the relevant literature.^85^ Neisser retorts in the same issue, immediately following Hansen’s article, labelling Hansen’s criticism as unobjective and a personal attack, asserting to know no sensible reason for Hansen’s behaviour. He could only assume that Hansen felt offended because Neisser contributed to research on leprosy and had an opinion differing from Hansen’s. Neisser notes that Hansen had consistently adopted an unfriendly tone since Neisser’s first publication on the topic, despite Neisser’s acknowledgement of Hansen’s contribution. He then sets out to belittle Hansen’s results: he failed to identify the observed micro-organism as a bacterium, Hansen’s first publication was overlooked, and his second publication added no new information. Then Neisser emphasises his own merits as the first to identify the micro-organism as a bacillus and to give an elaborate description of its distribution in the body.^86^ He even invokes Virchow as a confirming authority, but Virchow had only confirmed Neisser’s success, not any priority.^87^

Hansen, apparently offended by Neisser’s attempt to prove leprosy’s contagious nature before him, attacked Neisser incessantly, seemingly in the guise of criticising his results but in effect describing him as an incapable scientist. Conversely, Neisser continually stresses to have honoured Hansen’s merits, just to belittle them and assert his own merits, and then laments Hansen’s malevolent behaviour against him.^88^ In retrospect Hansen’s and Neisser’s polite words of appreciation for each other that can be found in the sources become sour remarks laden with irony and sarcasm. Ultimately, while the question of who discovered the leprosy bacterium was never in doubt, a personal enmity between Hansen and Neisser persisted from Neisser’s 1879 publication until the Berlin conference of 1897.

This dimension of the relationship between Hansen and Neisser reveals a less flattering side of Hansen, as he also engaged in similar attacks against other colleagues. When the editorial board of a Norwegian journal appealed to its contributors to abstain from personal remarks, Hansen interpreted this as a reprimand for his earlier articles in the journal, just to repeat the very same comments.^89^

## Conflict in context

The so-called Hansen-Neisser controversy was not a scientific controversy in the strict sense. It was no prolonged dispute about scientific methods, findings, or interpretations, nor did it influence political decisions regarding the treatment of leprosy as a public health issue. Just as it was not a controversy in the strict sense, the leprosy conference in Berlin in 1897 did not play a role in resolving it. Rather than a controversy, the affair was a professional rivalry and personal conflict centred on the competition to prove that *Mycobacterium leprae* caused leprosy. As such, the conflict between Hansen and Neisser was not unusual. Many other conflicts in medical history involved similar rivalries over priority and personal animosities. As previously indicated, the Hansen-Neisser conflict was not about priority. As priority can be difficult to determine, it is sometimes as important to be the first in the race to publish results as being the first to make an observation or development. In this regard, Hansen clearly succeeded.

However, such controversies extend beyond being first. As with the rivalry between the British physicians, Charles Clay and Thomas Spencer Wells, competing for priority in developing ovariotomy, it is also a contest to convince the scientific community.^90^ Here again, Hansen succeeded.

Establishing credibility proved to be a crucial factor for success. While Hansen had less credibility than Danielssen at the time of the discovery, Hansen had the advantage compared to Neisser seven years later. Hansen’s greater age, experience, and institutional authority conferred him more credibility than Neisser, who was younger and less established at the time.

Priority disputes can also exhibit traits of national antagonism. While this may not have been a prominent factor between Hansen and Neisser, the conflict between Pasteur and Koch serves as a typical example. Pasteur and Koch competed not for priority per se; rather they were vying for recognition of the importance of their respective own work.^91^ Each sought to downplay the achievements of the other while highlighting their own, a pattern that similarly applies to the Hansen-Neisser conflict.

While conflicts of this kind often are rooted in the egos of the disputants, these feuds can extend to the supporters of each scientist, continuing the conflict beyond its settlement.^92^ Concerning priority this was also the case in the so-called Hansen-Neisser controversy, when Czaplewski revived a matter that already had been abandoned two decades before. His statements show an undeniable nationalistic dimension. Also, Vogelsang and Irgens functioned as supporters when defending their idol Hansen against the alleged theft of his discovery by Neisser. While national sentiments – or, more accurately, local patriotism – may have played a role in this context, the influence was likely minimal. This reflects an older, rather hagiographic style of scientific biography that tends to celebrate heroic scientists, portraying them as strictly neutral and objective while downplaying the personal and political dynamics of science.^93^ However, it became evident from the previous discussion of the enmity that Hansen’s personality significantly impacted his behaviour as a scientist.

There are also parallels in how mistranslations have shaped the perception of science. Possibly the most famous stems from astronomy. In 1877, the Italian astronomer Giovanni Schiaparelli observed markings on the surface of Mars. He referred to them as ‘*canali*’, without clarifying whether they should be understood as artificial or as natural.^94^ The Italian term ‘*canali*’ was mistranslated into English as ‘canals’, suggesting that the observed features were the product of engineering efforts.^95^ Initially, Schiaparelli’s observations garnered little public interest and faced scepticism among scientists.^96^ However, in 1895, the wealthy amateur astronomer Percival Lowell popularised this interpretation, much to the disapproval of professional astronomers. He argued that the ‘canals’ were artificial, implying the presence of intelligent life on Mars.^97^ Doubts about the observations were expressed as early as 1882, attributing them to optical illusions. Eventually, the existence of any ‘*canali*’ was rejected in 1909.^98^ Nonetheless, Lowell’s writings had a significant impact on public perception of Mars.

When comparing this example with the so-called Hansen-Neisser controversy, interesting parallels emerge. Both cases begin with a degree of ambiguity, are initially shaped by mistranslations, and are ultimately popularised through widespread dissemination – all by different individuals. In the case of the so-called Hansen-Neisser controversy, the ambiguity arises from Hansen’s use of the term ‘bypassing’. Vogelsang mistranslates Neisser’s expression ‘widely neglected’ as ‘hitherto not observed’, thereby transforming the ambiguity of the use of ‘bypassing’ in Hansen’s memoirs into a challenge to Hansen’s status as the discoverer. This notion is further popularised as intellectual theft by Irgens. In Schiaparelli’s case, the ‘*canali*’ remain ambiguous and are mistranslated into artificial structures. Ultimately, the amateur Lowell popularised the idea that these features were the handiwork of intelligent Martian inhabitants.

Another notable instance of the influential popularisation of a scientific error by a non-professional is the misconception that people in the Middle Ages believed the Earth was flat. This notion gained traction following Washington Irving’s partially fictitious biography of Christopher Columbus, published in 1828 which continued to be widely accepted.^99^

## Conclusion: a debunked myth, a tie, and a beneficial conflict

The objective of this article was to examine how the narrative about the Hansen-Neisser controversy, which contradicts the primary sources, emerged and to describe the nature of the conflict that nonetheless existed between Hansen and Neisser. In this regard, the article contributes to the field of controversy studies and challenges established hagiographic narratives within the historiography of science.

This study demonstrates that there was no controversy regarding who first observed *Mycobacterium leprae.* This honour has consistently been attributed to Hansen. Nevertheless, Neisser took advantage of Hansen’s help, the tissue samples from Bergen, the information about Hansen’s results, and his failed attempts to stain the bacteria. Although the sources do not provide evidence, it is plausible that Hansen hoped or even expected Neisser to solve his problems and provide him with the desired information. Neisser’s conduct – publishing without consulting Hansen – was rude but not fraudulent. He never claimed the first observation for himself and consistently referred to Hansen. Therefore, there was no attempt of bypassing in terms of discovery.

As this analysis reveals, the erroneous perception of a controversy originated from incorrect translation and interpretation of sources. This perception was subsequently popularised by influential authors, evolving into a reified narrative akin to modern myths, such as the misconception that people in the Middle Ages believed the Earth was flat.

This case underscores the necessity of a critical approach to historical narratives, particularly those that are overly simplistic or present clear-cut hero-villain dichotomies. Technical skills, including traditional source criticism and language proficiency, are essential for analysing such narratives. This study has highlighted potential consequences of the misconception of historiography as a straightforward activity that anyone can engage in, even without the requisite training in the mentioned skills that are crucial in history, but not necessarily in other academic disciplines.

Furthermore, the article demonstrates that a conflict existed between Hansen and Neisser comprising two aspects: a professional rivalry and a personal enmity. The professional rivalry centred on the competition to prove that *Mycobacterium leprae* was the contagion that caused leprosy. Neither Hansen nor Neisser succeeded in this regard and even the proof for the first Koch postulate was completed by another physician. Although Neisser’s attempts failed, he can be accused of trying to bypass Hansen in terms of proof.

As this study has shown, the competition led to a second aspect of the conflict: personal enmity. Contrary to claims in literature, Neisser’s first publication did not aim to discredit Hansen or to diminish his result. The tone changed only after Hansen portrayed him as an incapable scientist. Neisser then started to belittle Hansen’s achievement while attempting to underline his own merits, which over time appeared increasingly desperate. Whereas Hansen disguised his attacks as critique veiled in academic rhetoric, Neisser consistently reacted offended and more direct, accusing Hansen of malicious intent and envy.

The rivalry, while ultimately unproductive in resolving the scientific question at hand, nevertheless served as a catalyst for further research. When Hansen got stuck, Neisser succeeded. Neisser’s less visible publication provoked Hansen to publish in a prestigious and renowned journal, which in turn paved the way for Neisser’s later publications. But it also created a lasting personal enmity between Hansen and Neisser.

## Footnotes

1

Thomas Martin Vogelsang , ‘Gerhard Henrik Armauer Hansen 1841-1912. The discoverer of the leprosy bacillus. His life and his work’, International Journal of Leprosy 46, 3 (1978), 257–332 365786. Lorentz Müller Irgens , ‘Hansen 150 Years After His Birth, the Context of a Medical Discovery’, International Journal of Leprosy 30, 3, (1992), 466–469 1474285. Lorentz Müller Irgens et al. (eds.), Lepra (Førde: Selja forlag, 2006). Johanne-Margrethe Patrix , Gerhard Armauer Hansen: leprabasillens oppdager (Bergen: Eide Forlag, 1997).

2

Gerhard Henrik Armauer Hansen , Livserindringer og betragtninger (Kristiania: H. Aschehoug & Co, 1910), 94 .

3 Vogelsang, *op. cit.* (note 1), Irgens *op. cit.* (note 1), Irgens et al. *op. cit.* (note 1), Patrix, *op. cit.* (note 1).

4

Steven Shapin , Never Pure (Baltimore: The Johns Hopkins University Press, 2010), 4f.

5

H. Tristram Engelhardt and Arthur L. Caplan (eds.), Scientific Controversies (Cambridge: Cambridge University Press, 1987).

6 Hansen, *op. cit.* (note 2), 93-94.

7

Eugen Czaplewski , ‘Albert Neisser und die Entdeckung des Leprabazillus’, Archiv für Dermatologie und Syphilis,124 (1917), 513–530. 523. Gerhard Henrik Armauer Hansen, Undersøgelser angående spedalskhendes årsager, *Norsk Magasin for Lägevidenskaben*, 9. Hefte, 3. Rekke, 4. Bind, Christiania 1874.

8

GL Fite and Herbert Windsor Wade , ‘The Contribution of Neisser to the Establishment of the Hansen Bacillus as the Etiologic Agent of Leprosy and the So-called Hansen-Neisser Controversy’, International Journal of Leprosy,23, 4 (1955), 418–427. 41813345503 .

9 Fite and Wade, *op. cit.* (note 8), 424.

10
*Ibid.*, 419.

11
*Ibid.*, 426.

12

Thomas Martin Vogelsang , ‘The Hansen-Neisser Controversy 1879–1880’, International Journal of Leprosy, 31, 1 (1963), 74–80. 7514059537 .

13 Vogelsang, *op. cit.* (note 12), 76.

14
*Ibid.*, 72.

15

Thomas Martin Vogelsang , G. H. Armauer Hansen (Oslo: Gyldendal, 1968), 74 .

16 Vogelsang, *op. cit.* (note 1), 313.

17 Patrix, *op. cit.* (note 1), 243, 250, 261.

18

Lorentz Müller Irgens , ‘Kort historikk vedrørende lepra og lepraforskning i Norge’, Nordisk Veterinærmedicin, 22, suppl 2 (1970), 23–29. 27. Lorentz Müller Irgens , ‘The Discovery of Mycobacterium leprae: A Medical Achievement in Light of Evolving Scientific Methods’, The American Journal of Dermopathology, 6, 4 (1984), 337–343. 3426388392. Irgens, *op. cit.* (note 1), 468. Irgens et al., *op. cit.* (note 1), 38.

19 Magnus Vollset, *Fra Lidelse til Trussel. Spedalskheten i Norge på 1800-tallet*, Master thesis (Bergen: University of Bergen, 2005).74. Magnus Vollset, *Globalizing Leprosy. A Transnational History of Production and Circulation of Medical Knowledge, 1850s-1930s*, PhD thesis (Bergen: University of Bergen, 2013), 11, 234.

20 Czaplewski, *op. cit.* (note 7). Fite & Wade, *op. cit.* (note 8). Vogelsang, *op. cit.* (note 12). Irgens, *The Discovery*, op. *cit.* (note 18).

21 Vollset, *Globalizing Leprosy. op. cit.* (note 19), 234.

22 Reinaldo Guilherme Bechler, *Leprabekämpfung und Zwangsisolierung im ausgehenden 19. und frühen 20. Jahrhundert: wissenschaftliche Diskussion und institutionelle Praxis*, PhD thesis (Würzburg: University of Würzburg, 2009), 103.

23 Bechler, *op. cit.* (note 22), 104, 105.

24
*Ibid.*, 105.

25

Thomas Ploemacher et al., ‘Reservoirs and Transmission Routes of Leprosy; A Systematic Review’, PLoS Neglected Tropical Diseases, 14, 4 (2020), 1–27, 1710.1371/journal.pntd.0008276PMC720531632339201.

26 Ploemacher et al., *op. cit.* (note 25), 8.

27

Linda Adams , ‘Susceptibility and Resistance in Leprosy: Studies in the Mouse Model’, Immunological Reviews, 301, 1 (2021), 158 10.1111/imr.12960PMC825254033660297.

28 Ploemacher et al., *op. cit.* (note 25), 12.

29 Adams, *op. cit.* (note 27), 158, 160.

30 Ploemacher et al., *op. cit.* (note 25), 16-17.

31

Albert Neisser , ‘Ueber die Aetiologie des Aussatzes’, Jahres-Bericht der Schlesischen Gesellschaft für vaterländische Cultur, 57 (1880), 65–72. 66, 71. Albert Neisser , ‘Weitere Beiträge zur Aetiologie der Lepra’, Virchows Archiv, 84 (1881), 514–542. 516. Heinrich Auspitz et al., *Handbuch der Hautkrankheiten.* V. 14 (in 2 pts.) of the Handbuch der speciellen Pathologie und Therapie/edited by Hugo Wilhelm von Ziemsen, (Leipzig: Vogel, 1883), 645. Hugo Wilhelm von Ziemsen , Handbook of Diseases of the Skin (New York: Wood & Co., 1885), 318 .

32 Hansen, *op. cit.* (note 2), 94.

33 Fite & Wade, *op. cit.* (note 8), 424.

34
*Ibid.*, 426.

35 Vogelsang, *op. cit.* (note 12), 76 f.

36 Neisser, *Weitere Beträge, op. cit.* (note 31), 516.

37 Vogelsang, *op. cit.* (note 15), 74.

38 Vogelsang, *op. cit.* (note 1), 289.

39 Irgens, *Kort historikk, op. cit.* (note 18), 27.

40 Patrix, *op. cit.* (note 1), 242, 250, 261. Irgens, *The Discovery*, *op. cit.* (note 18), 342.

41 Fite and Wade, *op. cit.* (note 8), 422.

42
*Mittheilungen und Verhandlungen der Internationalen Wissenschaftlichen Lepra-Conferenz zu Berlin im October 1897*, Bd 2. (Berlin: Hirschwald, 1897). 190 f.

43
*Ibid.*, 25.

44
*Mittheilungen und Verhandlungen der Internationalen Wissenschaftlichen Lepra-Conferenz zu Berlin im October 1897*, Bd 1. (Berlin: Hirschwald, 1897), 120-126.

45 Aloys Pollender, Microscopische und microchemische Untersuchung des Milzbrandblutes, so wie über Wesen und Kur des Milzbrandes*. Vierteljahrsschrift für gerichtliche und öffentliche Medicin.* Bd. 8 (1855), 103–114 (Berlin: Hirschwald 1855). Casimir Davaine, Recherches sur les infusoires du sang dans la maladie connue sous le nom de sang de rate. *Comptes rendus hebdomadaires des seances de l’Académie des Sciences.* Bd. 57, (1863), 220-223, 351-353, 386-387.

46 Robert Koch, Die Ätiologie der Milzbrand-Krankheit, begründet auf die Entwicklungsgeschichte des Bacillus Anthracis. *Cohns Beiträge zur Biologie der Pflanzen*, Bd. II, Heft 2, p 227, (Breslau: Kerns Verlag, 1876)

47

Robert Koch , Untersuchungen über die Ätiologie der Wundinfektionskrankheiten (Leipzig: F. C. W. Vogel, 1878). Robert Koch , Über die Ätiologie der Tuberkulose. Verhandlungen des Kongresses für innere Medizin. Erster Kongress (Wiesbaden: Verlag von J. F. Bergmann, 1882).

48

Friedrich Loeffler , Untersuchung über die Bedeutung der Mikroorganismen für die Entstehung der Diphtherie beim Menschen, bei der Taube und beim Kalbe. Mittheilungen aus dem Kaiserlichen Gesundheitsamte, 2 (1882), 421–499. 424.

49 Jakob Henle, *Von den Miasmen und Kontagien und von den miasmatisch-kontagiösen Krankheiten* (1840) (Leipzig: Barth, 1910), 52.

50

Edwin Klebs , Ueber die Umgestaltung der medicinischen Anschauungen in den letzten drei Jahrzehnten (Leipzig: Vogel, 1878), 41–45 .

51

Robert Koch , Die Ätiologie der Tuberkulose (Berlin: Hirschwald, 1884).

52 Hansen’s letter of defence 24.05.1880, Statsarkivet Bergen ICC 133 99-1880.

53

Gerhard Henrik Armauer Hansen , ‘Einige Bemerkungen über die anästhetische Form des Aussatzes’, Vierteljahresschrift für Dermatologie und Syphilis, 10 (1883), 557–560, 559.

54

Gerhard Henrik Armauer Hansen , ‘Bacillus Leprae’, Virchows Archiv, 79 (1880), 32–42. 40. Gerhard Henrik Armauer Hansen , ‘Studien über Bacillus leprae’, Virchows Archiv, 90 (1882), 542–548. 542.

55
*Ibid.*, ‘Bacillus Leprae’, 41. Hansen, *op. cit.* (note 2), 93.

56
https://www.dahw.de/organisation/geschichte/vorbilder/gerhard-armauer-hansen.html Koch, Robert. Fotostatkopi av brev til Gerhard Armauer Hansen, d. 20. 7. 1879. Ms 1732-b, Manuskript- og librarsamlingen, Universitetsbiblioteket i Bergen.

57 Neisser, *Weitere Beiträge, op. cit.* (note 31), 516.

58
*op. cit.* (note 56).

59

Gerhard Henrik Armauer Hansen , ‘Bacillus Leprae’, Nordiskt medicinsk Arkiv, 3 (1880), 1–10 . Hansen, ‘Bacillus Leprae’, *op. cit.* (note 54). Gerhard Henrik Armauer Hansen , ‘The Bacillus of Leprosy’, *Quarterly Journal of Microscopic Sciences*, New Series, 20 (1880), 92–102 . Gerhard Henrik Armauer Hansen , ‘Bacillus Leprae, études sur la bactérie de la lèpre’, Archive de Biologie, 1, (1880), 1–16 .

60

Gerhard Henrik Armauer Hansen , ‘On the Etiology of Leprosy’, The British and Foreign Medico-Chirurgical Review, 55 (1875), 459–489 PMC515787230162848.

61 Neisser, *Weitere Beträge, op. cit.* (note 31), 516.

62
*op. cit.* (note 52).

63
*op. cit.* (note 56).

64 Neisser, *Weitere Beiträge, op. cit.* (note 31), 533.

65 Auspitz, *op. cit.* (note 31), 643, 653.

66
*op. cit.* (note 44), 1.

67 Neisser, *Weitere Beiträge, op. cit.* (note 31), 533.

68 Hansen, *Studien, op. cit.* (note 54), 547. Gerhard Henrik Armauer Hansen , ‘Die Aetiologie und Pathologie der Lepra’, Vierteljahresschrift für Dermatologie und Syphilis, 11, 3-4 (1884), 317–336. 322.

69 Auspitz, *op. cit.* (note 31), 645. Ziemsen, *op. cit.* (note 31), 318.

70

Eduard Arning , ‘Ueber das Vorkommen des Bacillus leprae bei Lepra anaesthetica sive nervorum’, Virchows Archiv, 97, 1 (1884), 170–171 .

71
*op. cit.* (note 42), 40, 41.

72 Bechler, *op. cit.* (note 22), 106.

73
*op. cit.* (note 52).

74 Bechler, *op. cit.* (note 22), 118.

75
*Ibid.*, 109. Fite and Wade, *op. cit.* (note 8), 424.

76 Bechler, *op. cit.* (note 22), 104.

77 Fite and Wade, *op. cit.* (note 8), 420.

78
*Ibid.*, 424.

79 Hansen, ‘Bacillus Leprae’, *op. cit.* (note 54), 41f.

80 Hansen, ‘Die Aetiologie’, *op cit.* (note 68), 321.

81 Hansen, ‘Studien’, *op. cit.* (note 54), 542.

82
*Ibid.*, 544.

83

Gerhard Henrik Armauer Hansen and Carl Looft , Leprosy: In Its Clinical and Pathological Aspects (Bristol: Wright & Co., 1895), 42 .

84
*op. cit.* (note 42), 43.

85 Hansen, *op. cit.* (note 53), 557.

86

Albert Neisser , ‘Erwiderung auf den vorstehenden Aufsatz von Armauer Hansen in Bergen’, Vierteljahresschrift für Dermatologie und Syphilis, 10 (1883), 560–565 . 564f.

87

Rudolf Virchow , ‘Krankheitswesen und Krankheitsursachen’, Virchows Archiv, 79 (1880), 185–228. 208.

88 Neisser, *op. cit.* (note 86), 564.

89

Gerhard Henrik Armauer Hansen , ‘Den nye Lov om Spedalskhed’, Medicinsk Revue, 2 (1885), 351–364. 351.

90

Sally Frampton , ‘Patents, Priority Disputes and the Value of Credit: Towards a History (and Pre-History) of Intellectual Property in Medicine’, Medical History, 55 (2011), 319–324. 32221792254 10.1017/s0025727300005330PMC3143866.

91

Agnes Ullmann , ‘Pasteur-Koch: Distinctive Ways of Thinking about Infectious Diseases’, Microbe, 2, 8 (2007), 383–387. 386.

92

Robert K Merton , ‘Priorities in Scientific Discovery’, American Sociological Review, 22, 6 (1957), 635–659. 638.

93 Shapin, *op. cit.* (note 4), 5, 10.

94

Robert Crossley , Imagining Mars: A Literary History (Middletown: Wesleyan University Press, 2011), 67 .

95 Crossley, *op. cit.* (note 94), 68.

96
*Ibid.*, 69.

97
*Ibid.*, 106, 117.

98
*Ibid.*, 69, 121.

99

Jeffrey Burton Russel , Inventing the Flat Earth: Columbus and the Modern Historians (New York: Praeger, 1991), 51f.

